# Real-time organ perfusion monitoring of human kidney transplants using *ex vivo* normothermic perfusion and reflectance spectroscopy

**DOI:** 10.1098/rsos.242008

**Published:** 2025-03-12

**Authors:** P. Chandak, D. P. Bennett, B. L. Phillips, R. Uwechue, N. Kessaris, B. J. Hunt, C. J. Callaghan, A. Dorling, W. Hayes, N. Mamode, J. C. C. Day

**Affiliations:** ^1^ Transplant, Renal and Urology Directorate, Guy’s and St Thomas’ NHS Foundation Trust, Guy’s Hospital, Great Maze Pond, London, UK; ^2^ Department of Inflammation Biology, Centre for Nephrology, Urology and Transplantation, School of Immunology and Microbial Sciences, King’s College London, London, UK; ^3^ Interface Analysis Centre, HH Wills Physics Laboratory, School of Physics, University of Bristol, Bristol, UK; ^4^ Department of Nephrology and Transplantation, Great Ormond Street Hospital for Children NHS Foundation Trust, London, UK; ^5^ Thrombosis and Vascular Biology Group, Rayne Institute, Guys and St Thomas’ NHS Foundation Trust and King’s Health Partners, St Thomas’ Hospital, London, UK

**Keywords:** human transplant, reflectance spectroscopy, machine perfusion, perfusion monitoring

## Abstract

Transplantation is the standard treatment for end-stage kidney disease but carries with it a non-trivial risk of post-operative complication. There is a need for a continuous, real-time, not additionally invasive method of monitoring organ perfusion. We present an approach to allograft perfusion monitoring using a human kidney model using *ex vivo* normothermic perfusion (EVNP) and custom spectroscopic optical reflectance probes. Five discarded human kidneys underwent EVNP, spectroscopic measurement and were subjected to perfusion compromising events (rejection, thrombosis or haemorrhage). Oxygenated and deoxygenated haemoglobin spectra were fitted to the spectra acquired from the kidneys in order to estimate the oxygen saturation. Average oxygen saturations before the perfusion compromising events were estimated to be higher than after (or similar in the control cases). Changes in oxygen saturation estimated from measurements made continuously were synchronized well with changes in renal blood flow index measurements. This proof of concept study proves promising in identifying a technique for continuous monitoring of perfusion and oxygenation of a transplanted kidney *in vivo* with minimal additional invasiveness.

## 1. Introduction

Transplantation is the standard treatment for end-stage kidney disease and provides improved patient survival and quality of life compared to dialysis [1–3]. Several challenges may arise following implantation surgery including inadequate organ perfusion, a potentially devastating, recognized post-operative complication with a variety of causes.

These include thrombosis, rejection and systemic hypotension owing to haemorrhage. Kidney graft thrombosis can complicate up to 4−18% of paediatric kidney transplants in the early post-operative period. Younger children (<20 kg) receiving an adult-sized donor kidney are particularly susceptible owing to vessel calibre mismatch and a lower cardiac output that may not adequately perfuse the organ [4–11].

Currently, clinical and laboratory parameters, such as changes in serum creatinine level and urine output, are used to monitor post-operative transplant graft perfusion in conjunction with Doppler ultrasound (DUS) [12,13]. In an acute setting when there is a strong clinical suspicion of thrombosis or poor allograft perfusion (characterized by pain, reduced urine output and a rising serum creatinine level), DUS and laboratory parameters may not adequately reflect the dynamic changes in the transplanted organ’s perfusion state in real time. Additionally, DUS and laboratory analysis may be subject to delay owing to processing time and availability of personnel (particularly out of hours), and in the case of DUS, may be equivocal owing to the subjective interpretation of the images.

These non-invasive investigations may delay the diagnosis of any catastrophic vascular complications, and despite urgent surgical intervention, organ salvage may be futile owing to irreversible graft injury resulting in transplant loss. There is, therefore, a need for a more informed, dynamic, real-time, minimally additionally invasive method of continuous measurements of kidney allograft perfusion that may allow for more timely graft salvage interventions. We present a novel approach to this problem by detecting changes in real-time kidney allograft perfusion in an experimental human kidney model using *ex vivo* normothermic perfusion (EVNP), a bypass machine technology and custom-designed spectroscopic reflectance probes.

## 2. Experimental methodology

### 2.1. Clinical model: *ex vivo* normothermic perfusion of discarded human kidneys

**Figure 1.**
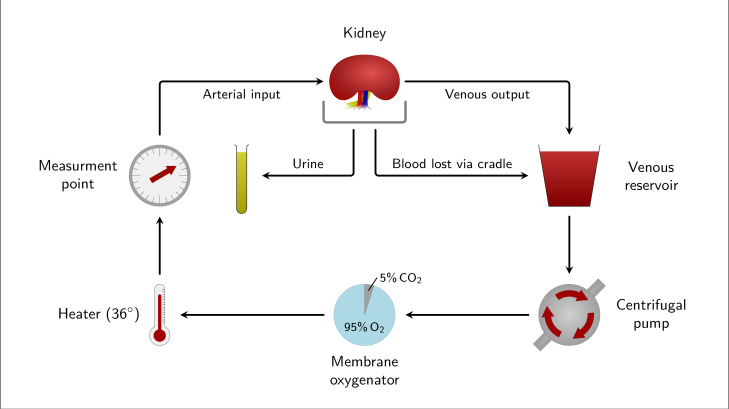
Schematic representation of EVNP (adapted from [14]). Venous reservoir primer: O^−^ red blood cells, heparin, Ringer’s lactate, dexamethasone, mannitol, NaHCO_3_ , prostacyclin, dextrose, insulin, and nutrients. Measurement point includes three-way tap for arterial blood gas samples, pressure transducer, and flow probe.

EVNP (figure 1) is a controlled perfusion technique based on paediatric cardiopulmonary bypass technology offering organ preservation, pre-conditioning and viability assessment, prior to transplantation. The technique involves perfusing an organ with warm, oxygenated, plasma-free red blood cell-based solutions allowing potential restoration of function, in addition to assessment of organ perfusion characteristics [14–18]. Specifically, EVNP allows the physiology of the kidney to be manipulated and maintained [19–23] and the delivery of pharmacological and cellular interventions to model a specific disease [24–34]. Furthermore, EVNP allows measurement of kidney function, including real-time blood flow, organ vascular resistance, urine output and biochemical markers [35,36]. This allows kidney performance during EVNP to be scored, which has been validated in human kidneys for transplantation [37].

Our study uses an experimental EVNP model using discarded human kidneys to simulate clinical conditions that may result in inadequate organ perfusion, namely rejection, thrombosis and haemorrhage/systemic hypotension. We proceeded to use EVNP to develop a clinically relevant translational model that allowed us to determine the efficacy of spectroscopic measurements in detecting perfusion changes within the donor kidney in real time that would result from the three clinically relevant scenarios.

### 2.2. Spectroscopic model: StO_2_ estimation from reflectance probe measurements

**Figure 2.**
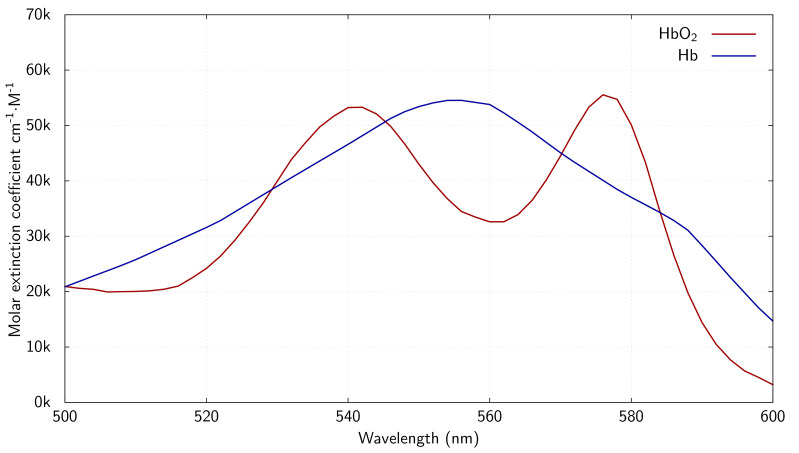
Oxygenated (red) and de-oxygenated (blue) haemoglobin absorption spectra at wavelengths 500 nm to 600 nm [38,39].

To estimate the oxygen saturation (StO_2_) of a perfused kidney from spectroscopic measurements, we evaluated the known spectroscopic profile of the primary oxygen-carrying medium within the kidney: haemoglobin. Figure 2 depicts the characteristic extinction coefficients in water for oxygenated (HbO_2_) and de-oxygenated (Hb) haemoglobin species, respectively, at wavelengths 500–600 nm [38,39].

These haemoglobin extinction coefficients can be converted to light absorption spectra when multiplied by a spectroscopic light path length and the haemoglobin species’ concentration in its suspension medium (i.e. blood/perfusate). Equation (2.1) [40] describes this relationship mathematically:


(2.1) $$A(\lambda )=\left\langle L\right\rangle \cdot \left\{c_{Hb}\cdot \epsilon_{Hb}(\lambda )+c_{HbO_{2}}\cdot \epsilon_{HbO_{2}}(\lambda )\right\}\cdot \ln10+A^{b}(\lambda )$$

The absorption spectrum described (*A*) is a weighted combination of the haemoglobin species’ extinction coefficients ($\epsilon_{HbO_{2}}(\lambda )$, $\epsilon_{Hb}(\lambda )$) in terms of their concentrations in the blood ($c_{HbO_{2}}$, $c_{Hb}$) and the spectroscopic light path length (⟨L⟩). A background component ($A^{b}$) is included in the formulation and is formed of a polynomial equation in *λ*.

To convert reflectance spectra acquired from the surface of the perfused kidneys into absorption spectra, the assumption was made that the light not reflected into the probe was absorbed by the kidneys, as opposed to transmitted. Absorption spectra were inferred by calculating the absolute difference between the measured reflection spectra and the input light spectra. Subsequently, a non-linear fitting algorithm [41,42] was used to fit the absorption model defined in equation (2.1) to each of the inferred absorption spectra acquired from the kidneys. This was achieved by varying the haemoglobin species’ concentrations (and other parameters) in the model until the sum of the squared differences between the mathematical model and the spectral measurements (the residual) was minimized (least squares method). The relative proportions of the resultant haemoglobin species’ concentrations were then used to estimate an ${StO}_{2}$ for the organ [40]:


(2.2) $${\text{StO}}_{2}=\frac{c_{HbO_{2}}}{c_{Hb}+c_{HbO_{2}}}$$

### 2.3. Experimental procedures

Five discarded human kidneys (deemed unsuitable for use in clinical transplantation) were obtained via consent and ethical approvals from NHS Blood and Transplant (NHSBT) and the Renal Project Board (Guy’s and St Thomas’ Trust Research and Development Study registration RJ115/N033). The kidneys were deemed not suitable for clinical transplantation owing to either prolonged cold ischaemia, donor age, poor *in situ* cold perfusion at retrieval of the donor kidney and if there was evidence of malignancy in the donor kidney or other organs. Additional permissions were secured from our local hospital blood bank to use discarded human-packed red cell units and fresh frozen plasma for all perfusion experiments. All organs were accepted under approved consent NHSBT pathways with no further research and ethics approvals being required. Exclusion criteria for using organs for our experiments were severe tissue and vascular damage following organ retrieval at the donor hospital, very poor perfusion at retrieval after cold flush with evidence of thrombosis and the presence of multiple vessels that could not be cannulated for use with EVNP. In addition, those kidneys with high-risk blood-borne viruses including HIV and hepatitis C were excluded. All accepted kidneys were delivered to Guy’s Hospital via transport services and disposed after use according to Human Tissue Authority regulations.

All kidneys underwent standard EVNP as previously described by Nicholson & Hosgood [43] and depicted in figure 1 to determine suitability for experimental intervention. The criteria for a kidney proceeding to experimental intervention was defined as achieving a steady renal blood flow index (RBFi) over a period of 15 min (based on procedures from our previous work [44]). Three sets of experiments were performed, in which the simulated clinical conditions would result in inadequate transplant graft perfusion, namely rejection, thrombosis and a hypotensive state (e.g. owing to blood loss). The clinical EVNP circuit was adapted with a reduced unfractionated heparin stat dose from the conventional 3000 units used for clinical EVNP purposes [43] to 375 units, so as not to impede the simulated rejection intervention, but to still maintain circuit flow.

Within each experimental setting RBFi, total urine output and spectroscopic measurements were recorded as experimental parameters. Spectroscopic measurements were performed using an Ocean Optics reflectance kit consisting of a FLAME-S-VIS-NIR spectrometer, an HL-2000-HP-FHSA tungsten halogen light source and a hand-held reflection probe placed on the kidney surface. In later experiments, the hand-held reflectance probe was substituted for a custom-designed and fabricated reflectance probe. The custom probe consisted of a dual-core polymethyl methacrylate optical fibre with a chamfered end to direct the transmission and detection to the kidney surface.

**Figure 3.**
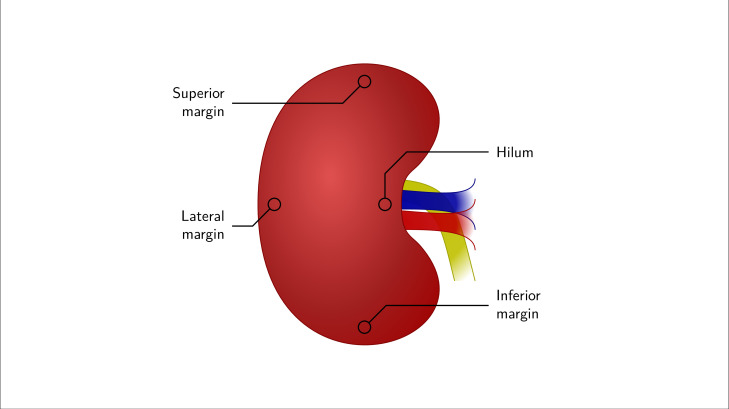
Schematic representation of hand held probe measurement points on kidneys K_A_, K_B_, and K_C_.

The initial spectroscopic measurement protocol used for kidney A (K_A_), kidney B (K_B_) and kidney C (K_C_) specified four measurement points of interest in the kidney: the hilum and the superior, lateral and inferior margins (figure 3). Reflectance spectra were acquired at these points at various stages of the perfusion cycles by placing the reflection probe lightly onto the kidney surface at each measurement point and activating the spectrometer. Reflectance spectra were acquired with an acquisition time of 5 s. The later spectroscopic measurement protocol used for kidney D (K_D_), and kidney E (K_E_) allowed reflectance spectra to be acquired repeatedly from one location on each of the kidneys using the custom probe. In the latter case, spectra were acquired with an acquisition time of 15 s. It was necessary to eliminate as much ambient light as possible while using the bespoke probe due to the exposed end. This was achieved by turning the theatre lights off. It is anticipated that ambient light will not be an issue *in vivo*.

#### 2.3.1. Simulation of rejection

Kidneys K_A_ and K_B_ were from the same donor. K_A_ was used to simulate severe antibody-mediated rejection (AMR) with K_B_ being a control. After reaching steady RBFi during EVNP, one unit of flash-frozen plasma (FFP; a source of complement and coagulation factors) with 600 µg of anti-Human Leucocyte Antigen class 1 IgG mouse monoclonal antibody (W6/32) were added to K_A_’s perfusate mix [44]. K_B_ underwent the same experimental conditions as K_A_, except a placebo antibody (normal saline) was used instead of W6/32. Haemodynamic machine parameters were recorded in 5 min intervals in both experiments. Reflectance spectra were taken at the four measurement points detailed above (the hilum and the superior, lateral and inferior margins) using the hand-held ‘end on’ reflectance probe. Spectra were acquired at each measurement point four times in the perfusion process, twice before the administration of FFP and W6/32 (or placebo in the case of K_B_), and twice after.

#### 2.3.2. Simulation of arterial thrombosis

Kidney K_C_ was subjected to the same experimental conditions applied to K_B_ (the control kidney) in the first set of experiments. As before, haemodynamic machine parameters were recorded at 5 min intervals. Reflectance spectra were taken at the four measurement locations described above. In the case of K_C_, spectra were acquired before perfusion had started while the kidney was being prepared to undergo EVNP and at five subsequent times during EVNP. At the end of EVNP, the pump flow was reduced from 100% to 0% in 25% reduction intervals, which gradually reduced the arterial inflow into the kidney, hence simulating the clinical scenario of arterial thrombosis. During this reduction, additional reflectance spectra were acquired at the superior margin sample point for each pump flow.

#### 2.3.3. (Inadvertent) simulation of haemorrhage/systemic hypotension

Kidneys K_D_ and K_E_ were originally intended to follow the same experimental method as kidneys K_A_ and K_B_. As such, K_D_ was subject to the same experimental conditions as K_A_ with K_E_ to act as the corresponding control. However, when EVNP in K_E_ was commenced, a significant volume of blood was lost through a small defect in the artery that was not identified during surgical preparation of the kidney prior to EVNP. The bleeding was abated by suturing the defect in the artery. The blood loss suffered by K_E_ provided an opportunity to evaluate the effect of acute blood loss on the perfusion characteristics of the organ, hence simulating a hypotensive clinical state owing to the loss of volume from the perfusion circuit. Reflectance spectra were acquired using the custom-designed and fabricated prototype probe that was secured to the surface of each kidney with polypropylene sutures. Spectra were acquired sequentially from each kidney at 15 s intervals with a 15 s acquisition time.

## 3. Experimental results

**Figure 4.**
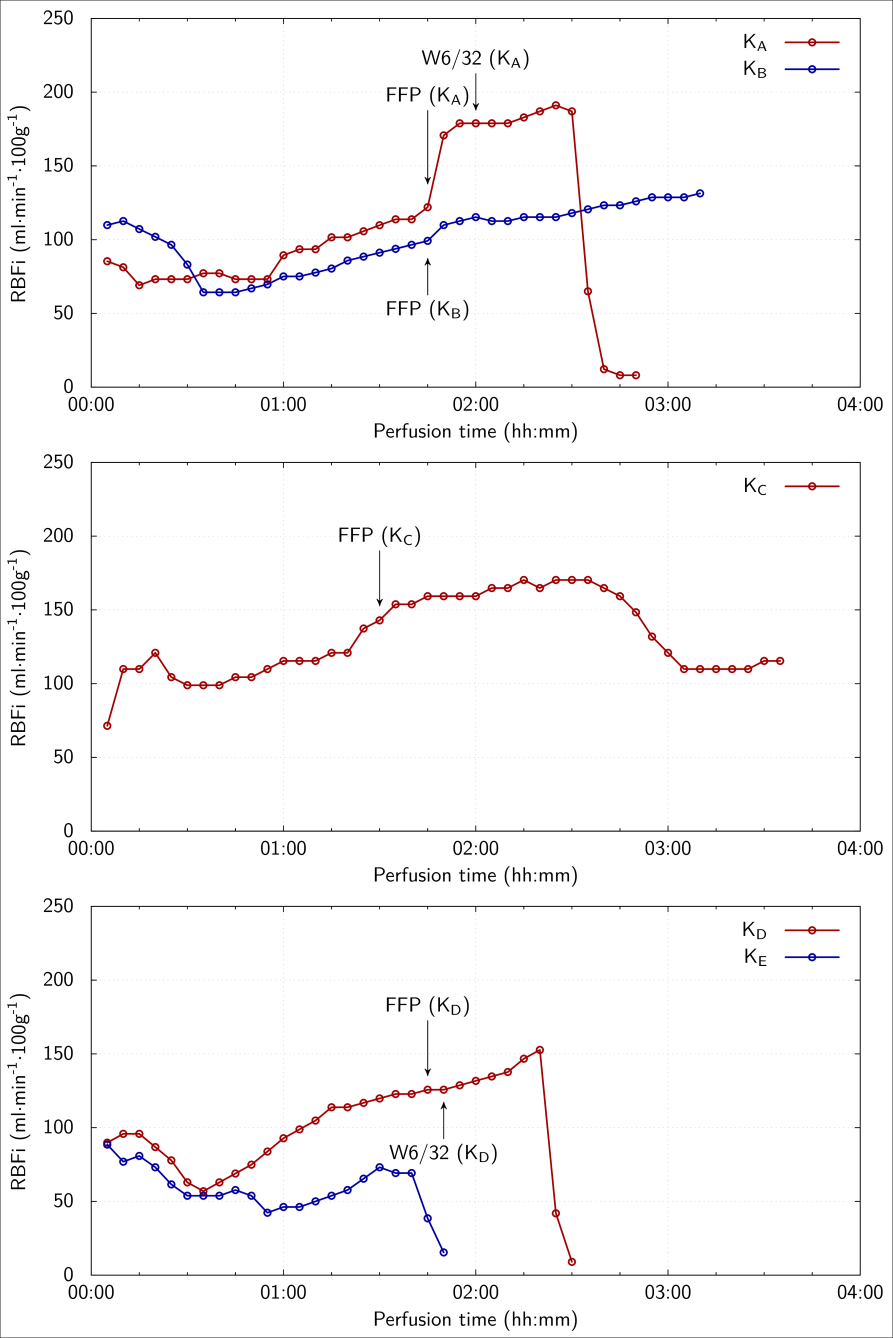
RBFi summary for all kidneys, grouped by donor (top K_A_ and K_B_, middle K_C_, and bottom K_D_ and K_E_). Applications of FFP and W6/32 to the perfusate during EVNP are indicated.

The demographics of the five discarded human kidneys are shown in table 1. All kidneys were from donation after circulatory death (DCD) donors. Cold ischaemia time is defined as the time from when the organ is perfused with cold preservation fluid within the donor at the time of retrieval to the time the organ commences warm perfusion. Figure 4 shows the RBFi during EVNP for each simulated clinical scenario, and table 2 shows the total urine output for each kidney for the duration of perfusion.

**Table 1.** Demographic data of the five discarded human kidneys. Cold ischaemia time (CIT) in (hh:mm).

kidney	experimental model	age	weight (g)	CIT	cause of death	discard reason
K_A_^a^	AMR	47	246	15:29	intracranial haemorrhage	gastric cancer
K_B_^a^	control for K_A_	47	373	19:44	intracranial haemorrhage	gastric cancer
K_C_	thrombosis	64	182	47:40	intracranial haemorrhage	renal cancer
K_D_^b^	AMR	67	334	26:00	respiratory failure	renal tumour
K_E_^b^	haemorrhage	67	260	30:45	respiratory failure	renal tumour

^a^ Kidneys originated from the same donor.

^b^ Kidneys originated from the same donor.

**Table 2.** Total urine output for each kidney.

kidney	urine output (ml)
K_A_	72
K_B_	69
K_C_	250
K_D_	79
K_E_	168

### 3.1. Simulation of rejection

K_A_ and K_D_’s experiment cycles were terminated by decompensation events (where the kidneys abruptly expelled and subsequently refused to accept the ingress of blood). This was characterized by the abrupt reduction in the RBFi from 30–40 min after the introduction of W6/32 into the perfusate (figure 4). Kidney K_B_ (control) maintained a steady RBFi throughout its experimental cycle. The estimated StO_2_ at the various measurement points and times for K_A_ and K_B_ are presented in tables 3 and 4. In the case of K_A_, the average estimated StO_2_ before the introduction of W6/32 is approximately 65%, while the average estimated StO_2_ after the introduction of W6/32 is approximately 53%. K_B_’s average estimated StO_2_ before the introduction of the placebo is approximately 92%, while the average estimated StO_2_ after the introduction of the placebo is approximately 93%, consistent with a steady RBFi with no decompensation. The variability between measurement locations on K_B_ is less pronounced than that seen in K_A_’s measurements.

**Table 3.** StO_2_ estimated from fitted oxygenated and deoxygenated haemoglobin concentrations at each measurement point for K_A_.

perfusion time	hilum	superior	lateral	inferior	average
00:30	0.834	0.804	0.635	0.426	0.675
01:40	0.464	0.743	0.759	0.746	0.678
02:05	0.474	0.750	0.445	0.436	0.526
02:40	0.366	0.688	0.599	0.506	0.540

**Table 4.** StO_2_ estimated from fitted oxygenated and deoxygenated haemoglobin concentrations at each measurement point for K_B_.

perfusion time	hilum	superior	lateral	inferior	average
01:00	0.873	0.834	0.991	0.923	0.905
01:35	0.895	0.960	0.950	0.929	0.934
02:00	0.895	0.949	0.978	0.941	0.941
02:35	0.917	0.885	0.962	0.873	0.909

Kidney K_D_ was the first kidney to have the custom-fabricated reflectance probe used. While using this probe on K_D_, ambient light was kept to a minimum where possible but was unavoidable at various periods during the EVNP perfusion cycles, in particular, at the start of EVNP while visually (qualitatively) assessing the kidneys’ perfusion quality and during the planned interventions involving the addition of FFP and W6/32 to the perfusate mixture. Additionally, it was necessary to adjust the positioning of the probe a number of times during the experiment because of slippage and rotation of the probe within the sutures used to secure it to the kidney. Owing to these difficulties, large changes in the consistency and quality of the acquired spectra manifested. The resultant spectra have been abandoned from an analysis perspective, and the results are limited to a learning experience regarding managing lighting changes, probe attachment and future probe design development.

### 3.2. Simulation of thrombosis

**Figure 5.**
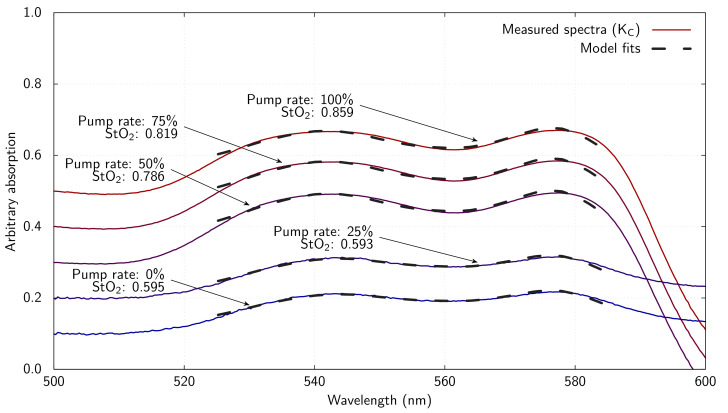
Inferred absorption spectra derived from reflection spectra acquired during EVNP pump step down with haemoglobin absorption model fits overlaid. Spectra are offset from one another vertically for clarity.

The estimated StO_2_ at various measurement points and times for K_C_ are presented in table 5, including those obtained during surgical preparation of the kidney prior to EVNP (approx. 50 min before perfusion began). K_C_’s average estimated StO_2_ is approximately 21% 50 min prior to EVNP and approximately 85% during EVNP. Additional spectra obtained from K_C_ at the end of the perfusion cycle while the EVNP pump flow was reduced in stages to simulate thrombosis are shown in figure 5, along with the absorption model fits overlaid. The spectra are shown with normalized magnitude and offset vertically from one another for clarity.

**Table 5.** StO_2_ estimated from fitted oxygenated and deoxygenated haemoglobin concentrations at each measurement point for K_C_.

perfusion time	hilum	superior	lateral	inferior	average
−00:50^a^	0	0	0.089	0.745	0.209
01:00	0.992	0.934	0.947	0.942	0.954
01:50	0.707	0.286	0.831	0.790	0.653
02:20	0.971	0.937	0.971	0.887	0.942
03:00	0.687	0.919	0.749	0.903	0.815
03:35	0.806	0.851	0.913	0.904	0.869

^a^ Spectra were acquired before EVNP started, during benching.

**Figure 6.**
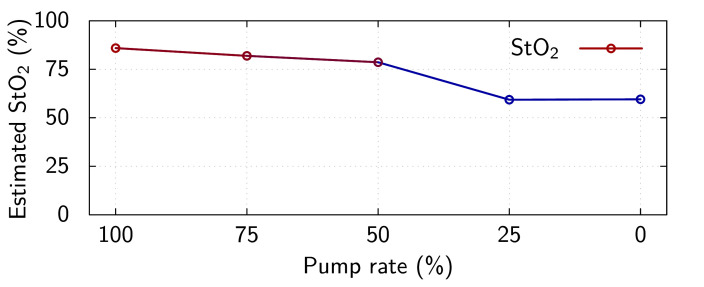
Oxygen saturation (StO_2_) estimated from fitted oxygenated (HbO_2_) and deoxygenated (Hb) concentrations at the superior margin of K_C_ for various EVNP pump rates.

Figure 6 and table 6 present the estimated StO_2_ calculated from the fitted haemoglobin concentration parameters for K_C_ at the various pump flow rates. The estimated StO_2_ level in K_C_ declines with the reducing pump flow, as expected, until the EVNP pump flow reaches 25%, where after the estimated StO_2_ level plateaus. The most abrupt change in estimated StO_2_ level occurs between the 50 and 25% pump rates.

**Table 6.** Summary of StO_2_ data shown in figures 5 and 6.

pump flow	StO_2_
100%	0.859
75%	0.819
50%	0.786
25%	0.593
0%	0.595

### 3.3. (Inadvertent) simulation of haemorrhage/systemic hypotension

**Figure 7.**
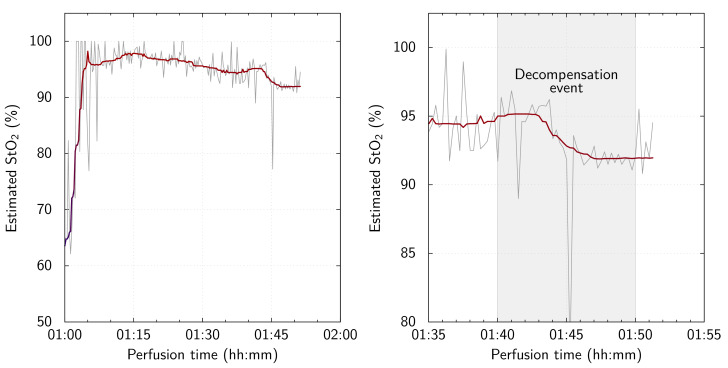
Continuously oxygen saturation (StO_2_) estimated from oxygenated and deoxygenated haemoglobin concentrations with time during K_E_’s perfusion cycle. Median smoothing of the StO_2_ is shown over the raw estimates. Left: the final hour of EVNP, shortly after the final repositioning of the reflectance probe on K_E_ . Right: the final 20 min of EVNP with the RBFi derived decompensation event window highlighted.

Like K_D_, K_E_ used the custom-fabricated reflectance probe and continuous spectrum acquisition methodology. While the ambient light level was kept more consistently reduced, it was necessary to illuminate the room and halt spectral measurements while attempting to abate K_E_’s unexpected arterial bleed. Additionally, despite success in preventing the probe from *twisting* when secured to the kidney, the probe continued to slip across the kidney surface (both abruptly and extremely slowly at various times, mainly owing to the stiffness of the cladding), which manifested as changes in the morphology of the acquired spectra. The probe was repositioned on the kidney surface several times until the position was stable. Approximately 1 h after EVNP was commenced, the probe’s position was judged to be satisfactory. Figure 7 shows the StO_2_ estimates with time during the final hour of EVNP for K_E_. K_E_’s experiment cycle was terminated by a spontaneous decompensation event. This was characterized by the abrupt reduction in the RBFi from 100 to 110 min after EVNP was commenced. The estimated StO_2_ dropped from approximately 95–92% in temporal synchronization with the drop in RBFi experienced by K_E_ (see figure 4).

## 4. Discussion

Herein we present a reproducible translational human kidney model of continuous real-time perfusion monitoring using EVNP and spectrographic measurements as a proof of concept. We have demonstrated the application of this model in three different experimental scenarios simulating clinical scenarios of antibody-mediated rejection, arterial thrombosis and hypotension, all of which may result in hypoperfusion of the organ at any stage in the post-operative period with the potential of graft loss.

### 4.1. Simulation of rejection

We present a model of acute severe rejection using W6/32 [44]. In this model, K_A_ suffered catastrophic reduction in blood flow 30–40 min post-infusion of the antibody, as expected. The control kidney, K_B_, for this experimental scenario maintained its blood flow. The (uncalibrated) estimates of the average StO_2_ calculated for K_A_ align with initial experimental expectations, i.e. the organ is measured to be less well-perfused on average after the introduction of W6/32 into the perfusate mixture. There was significant variation in estimated StO_2_ between K_A_’s measurement locations at each measurement time, however, which is attributed to variability in manual positioning of the hand-held probe, variability in the pressure applied to the organ surface by the probe and variability in the surface condition of the organ (moisture sheen, blood seepage, etc.), all of which affect the spectroscopic measurements being made. The estimates of the average StO_2_ levels for K_B_ appear significantly higher than those seen in K_A_. It is not clear if the difference in the general estimated StO_2_ level is owing to inter-organ variability or owing to the variability in the hand-held measurement procedure affecting the baseline of the measurements. The average StO_2_ calculated for K_B_ also aligns with initial experimental expectations, i.e. the organ is measured as similarly well-perfused on average before and after the introduction of the control placebo (saline).

### 4.2. Simulation of thrombosis

We created a model of the effect of arterial thrombosis on graft perfusion by controlled reduction of the EVNP pump flow. This was the first experimental scenario that provided a secondary enumeration of the kidney perfusion state (the pump flow). In this model, the estimates of the StO_2_ calculated for K_C_ are mostly aligned with initial experimental expectations, i.e. very low StO_2_ estimates pre-perfusion, and similarly well-perfused on average (excluding two outlier measurements) during perfusion. Additionally, the estimated StO_2_ levels during the post-EVNP pump flow reduction phase are very well aligned with initial experimental expectations, i.e. a linear reduction in estimated StO_2_ with linear reduction in EVNP pump flow until the mean arterial pressure fell below the perfusion threshold of K_C_ (between the 50 and 25% pump rates). Below this threshold, the circuit flow could not be maintained, and the estimated StO_2_ level plateaued, as expected with a static circuit.

### 4.3. (Inadvertent) simulation of haemorrhage/systemic hypotension

We created a model of systemic hypotension owing to blood volume loss that resulted from an arterial defect. Our initial expectations (formed after deciding to continue with the experiment after abating the bleed) were that a gradual reduction in perfusion, and subsequent decompensation of the organ would occur because of the sustained exposure to the reduced volume in the perfusion circuit. K_E_’s behaviour aligned with the latter of these expectations; however, the decompensation event occurred abruptly and was not preceded by an obvious gradual decline in the RBFi or in the continuous StO_2_ estimates. There was extremely good synchronization between the measured decline in RBFi and the continuous estimates of StO_2_ during the decompensation event.

## 5. Conclusions

Both vascular and non-surgical complications may adversely affect blood flow and micro-circulation within an organ. Early diagnosis and timely interventions in these situations are time-critical for graft salvage, as most thrombotic events occur within the first 7 days post-transplantation, particularly within the first 24−48 h [11,45–48]. In their study of 312 kidney transplants, Ammi *et al*. [49] report an incidence of 1.9% of transplant graft nephrectomy owing to arterial and venous thrombosis. In addition to vascular surgical complications, other pathologies may also adversely affect transplant perfusion. In a further study of 48 kidney transplant recipients, Wang *et al*. [50] reported that acute AMR is associated with a significant reduction in kidney transplant cortical perfusion when evaluated using power DUS. Although DUS is a non-invasive and non-toxic modality useful for assessing general global perfusion of the kidney and patency of the major blood vessels, it may not provide accurate information about distal (micro-circulation) perfusion and does not provide prolonged continuous, real-time monitoring (>24 h). DUS may also be limited in determining the aetiology of perfusion dysfunction in the immediate post-operative period [51]. Furthermore, DUS is operator-dependent and may be prone to technical and subjective interpretation errors. Critically, radiology personnel may not always be readily available out of hours, which may delay any diagnosis of hypoperfusion states in the transplanted kidney with devastating consequences [13,52–54]. In this regard, an alternative approach to real-time perfusion monitoring is required.

The importance of continuous perfusion monitoring applications has been previously alluded to [55]. Malakasioti *et al*. [56] report the correlation of near-infrared spectroscopy (NIRS) with DUS perfusion and haemodynamic parameters in stable paediatric transplant recipients and suggest this could be applied to continuous monitoring. Others have reported similar experiences with NIRS in the context of kidney transplantation [57–59] and in identifying acute kidney injury following infant cardiac surgery [60]. Thermal diffusion probes have been reported to provide continuous micro-circulatory monitoring of kidney cortical perfusion in a porcine model [61], followed by its clinical application in 30 kidney transplants [62]. Microdialysis catheters may also be used to provide dynamic monitoring of changes in the concentration of metabolites in tissues in different physiological states. In their porcine model of kidney transplantation, Fonouni *et al*. [63] reported that microdialysis can aid the early detection of vascular thromboses. Reduced glucose levels and increased lactate-to-pyruvate ratios with increased glycerol levels are indicators for the early detection of vascular complications post-kidney transplantation [63]. However, both microdialysis and thermal diffusion techniques rely on the insertion of probes into tissues with the potential for trauma and bleeding, and further validation studies may be warranted [47]. Reflectance spectroscopy is superior to implantable Doppler devices that have also been described to monitor free flaps [64]. Implantable Doppler techniques assess the main arterial and venous vessel blood flow only, whereas our reflectance probe measures organ tissue perfusion. In children receiving kidney transplants, perfusion of the kidney parenchyma requires assessment in real time as this may be compromised by issues that Doppler measurements of flow do not detect (e.g. vessel stenosis and suboptimal perfusion pressure). In addition, implantable Dopplers have not been adopted in clinical transplant practice owing to the practical difficulties of implanting and removing them.

Continuous monitoring of kidney allograft perfusion is critical post-transplantation where intervention maybe necessary for graft salvage. If DUS is unavailable or provides equivocal information regarding allograft perfusion characteristics, surgical exploration becomes mandatory. In acute circumstances, this may be too late for graft salvage. Continuous monitoring is expected to be most useful in transplanted kidneys that are at increased risk of thrombosis, such as those from elderly or very young donors, those having multiple vessels and those with prolonged cold ischaemia time from DCD donors. This is underlined by the observation that all of the negative outcomes which occurred for the kidneys in this study occurred abruptly. This proof of concept study proves promising in identifying an alternative, not additionally invasive, and non-nephrotoxic method for continuous monitoring of perfusion and oxygenation of a transplanted kidney *in vivo*. Clinical translation could be achieved through the inclusion of a suitable optical reflectance probe adjacent to or within the lumen of a wound drain that is routinely placed alongside the transplanted organ before abdominal closure. Further studies are required to evaluate the optimal probe design and attachment modality to minimize probe movement and optimize spectra quality in the post-operative period. Additionally, our data does not give an indication regarding any possible variation in the perfusion of the organ spatially and therefore does not inform on the appropriate probe position on the kidney (e.g. the hilum or the superior, lateral and inferior margins).

We expect that with refinement of the custom probe, it will be possible to eliminate operational variability in the acquisition of reflectance spectra and improve StO_2_ estimates, allowing the identification of sub-clinical haemodynamic changes within the kidney, which may have clinical significance in early graft salvage interventions. There is also additional scope to explore improvements on the continuous wave system (e.g. a frequency domain system), although the simple successful detection in negative differential of perfusion in real time during the critical 24−48 h after transplant may be a sufficient warning system. To our knowledge, this is the first study reporting the feasibility of continuous monitoring using reflectance probes and spectroscopy in a human kidney model using clinical-grade warm machine perfusion technology.

## Data Availability

The data can be accessed online [65].
